# Effects of pronatal policies on sexual and reproductive health services: a protocol for a scoping review

**DOI:** 10.1136/bmjopen-2025-115081

**Published:** 2026-04-15

**Authors:** Gitau Mburu, Alanna Galati, Doris Chou, Bela Genatra, James Kiarie, Victoria Boydell

**Affiliations:** 1 UNDP-UNFPA-UNICEF-WHO-World Bank Special Program of Research, Development and Research Training in Human Reproduction (HRP) Department of Sexual, Reproductive, Maternal, Child, Adolescent Health and Ageing, World Health Organization, Geneva, Switzerland; 2 Institute of Women’s Health, University College London, London, UK

**Keywords:** Health policy, Reproductive medicine, Pregnant Women

## Abstract

**Introduction:**

In the context of declining total fertility rates, many governments are seeking the optimal blend of policy interventions to encourage childbirth. Scholars have mapped how pronatal policy agendas are shaping social policies, yet there has been less attention on how such agendas shape health policies. This review will map and synthesize the evidence on how pronatal policies affect sexual and reproductive healthcare.

**Methods and analysis:**

A scoping review will be conducted using two searches. One search will identify relevant peer-reviewed papers in Medline, EMBASE, social policy and practice, global health and Web of Science databases. A second search will identify relevant grey literature from Overton and Policy Commons databases. Sources will be managed using Rayaan software and studies selected based on specified inclusion criteria. Data will be extracted using a customised extraction form, mapped and subsequently synthesised using narrative approach.

**Ethics and dissemination:**

This review will be disseminated through a peer-reviewed manuscript and conference presentations. Ethics and patient engagement are not required for a scoping review.

**PROSPERO registration number:**

CRD420251156155.

**STRENGTHS AND LIMITATIONS OF THIS STUDY**

- Insights from this review will identify a range of adaptations to declining total fertility rates and their varying impacts on sexual and reproductive health services, in an evolving policy landscape.
- Given the scarcity of subject headings related to pronatal issues and declining total fertility rates, the review will combine different literature sources to map the evidence.
- As this is a scoping review, quality assessment of included studies will not be conducted.

## Introduction

 Total fertility rates (TFRs) (which is the average number of children that a woman would have over their lifetime if the current age-specific fertility rates are maintained) are declining globally.^1^ In a practical sense, this means that the average number of children born within a specific area or period is declining, and people are increasingly having fewer children over their lifetime, often fewer children than they desire.^2^ TFR at or below the replacement level of 2.1 is an increasing global trend. Countries and territories with below replacement fertility (of 2.1) are predicted to increase from 103 (50.5%) countries in 2050 by 25% to 155 (76%) in 2050 and double to 198 countries (97.1%) by 2100.^3^

While declining TFR has pros and cons,^4^ declining TFR is perceived as a threat in some contexts, creating anxieties about a shrinking workforce, reduced productivity and existential concerns for the future.^5^ Based on these concerns, governments have implemented explicit or implicit pronatal policies; these are state-led policies that intentionally encourage people to have more children and increase the birth rate within a population. According to the World Population Policy database, the percentage of countries with pronatal policies has risen from 19% in 1976 to 28% by 2019.^6^

While many governments are seeking the optimal blend of pronatal policy interventions that are effective at the population level and socially acceptable to individuals, the implementation of these policies has inherent tensions. On the one hand, such policies ought to support self-determination and the achievement of individual fertility preferences, ensuring that the number of children a person has aligns with the number they want. Yet, on the other hand, some policies may be at odds with self-determination, insofar as they exacerbate unmet need for reproductive planning services and/or are otherwise experienced as coercive.

Pronatal policies that are aimed to help individuals achieve their reproductive goals vary widely and straddle many sectors. These policies support people to have the children they desire through a range of financial and social support initiatives. Examples are direct payments for having children, tax benefits, subsidised early years childcare and parental leave.^7^,^10^ At the same time, pronatal policies may actively restrict access to safe abortion and contraception services or promote unregulated fertility care. There is therefore a risk that, when faced with declining TFRs, some countries might adopt pronatal policies that restrict services related to sexual and reproductive health and rights (SRHR) and reduce reproductive autonomy, which includes the right to choose whether or not to have children, the timing and spacing of pregnancies and access to comprehensive sexual and reproductive healthcare.^11 12^

Commentators on this risk have emphasised that declines in TFR should not be used to limit or coerce utilisation of contraception, abortion or infertility services.^11 13^ Other researchers have indicated the need for countries to focus beyond demographic metrics and instead centralise reproductive rights.^14^ Yet, there is only sparse evidence of how pronatalist policies can positively or negatively impact an individual’s rights to access healthcare, especially sexual and reproductive services. To illustrate the potential impact of pronatal policies and to support countries in responding to declining fertility from a human rights approach, it is essential to document and synthesise existing empirical evidence on the impact of such policies on a range of SRHR areas.

To date, most literature reviews of the impact of pronatal policies have tended to focus on social and financial policies, for example, related to childcare, tax benefits and parental leave. Limited existing research has considered how pronatal policies affect SRH services and for whom. In addition, where this evidence exists, it is not comprehensively documented or synthesised. There is, therefore, a need for a systematic scope and synthesise existing evidence because (1) the evidence is scattered across a range of sources, (2) we do not know the extent or nature of this evidence and (3) we do not have empirical evidence of the impacts of pronatal policies on access and/or utilisation of SRH services. A comprehensive synthesis demonstrating whether, to what extent and what is the nature of the evidence on how pronatal policies affect SRH and their empirical impacts on sexual and reproductive services is lacking.

Given the above need, we will conduct a scoping review focusing on the pronatal policies related to sexual and reproductive healthcare services. Here we refer to both the Guttmacher-Lancet Commission^15^ and the International Conference on Population and Development^16^ definition of these services, which closely align. Given the broad scope of SRHR services, it is difficult to assess every single service which forms part of a comprehensive SRHR services within a health system. Consequently, this review will use tracer services, a subset of comprehensive SRHR services that can be used as proxy indicators to measure or assess the impact on overall SRH services. Specifically, we will use four tracers: abortion, contraception, preconception/antenatal and fertility care services, as these are well-documented areas that can potentially serve as proxies for wider SRHR services.

### Aim

The broad aim of this scoping review is to map the evidence on the nature, extent and impact of pronatal policies on SRH services.

### Specific objectives

This review will:

1. Identify the extent and nature of the evidence on whether pronatal policies (ie, policies intended to promote birth rates and population growth) affect selected tracer SRH services.
2. Identify the extent, range and nature of evidence on the relationship between pronatalist policies and selected tracer SRH services.
3. Identify key terms, constructs and theories that are defined and measured in the literature related to prenatal policies.
4. Identify disciplinary trends, study design, geographies, rationales, policy mechanisms, levels of implementation and reported outcomes of these policies in the literature.
5. Identify patterns in the evidence and knowledge gaps that can shape future research priorities.
6. Identify empirical examples of the impact of pronatal policies on tracer SRH services.

## Methods and analysis

### Framework

This scoping review is designed and reported according to the Preferred Reporting Items for Systematic Reviews and Meta-Analysis (PRISMA) extension for scoping reviews.^17^ PRISMA-Protocols checklist is attached as online supplemental file 1. A protocol of the analytical methods and inclusion criteria was registered with the International Prospective Register of Systematic Reviews database (PROSPERO; registration number 2025, CRD420251156155). Any deviations from this protocol will be disclosed and explained for transparency.

### Information sources and search strategy

Two search strategies will be employed. A librarian will be consulted to assist in refining the initial search strings and finalising the search terms to ensure the search returns relevant literature with the maximum scope and sensitivity. The first search will explore peer-reviewed literature on pronatal policies published in the last 50 years. The literature search will be conducted in the following electronic bibliographic databases, which focus on medical and health research: Medline, EMBASE, social policy and practice, global health and Web of Science. Specific search terms will be specified for each database. Search terms included are outlined in table 1.

**Table 1:** Search terms

SEARCH 1	pronatalis* OR pro-natalis* OR Population polic* OR low fertility OR declining fertility OR anti-abortion policies OR Population decline OR demographic crisis OR Sub-replacement fertility OR Below replacement fertility OR Shrinking population OR Ag?ing population OR population ag?ing OR depopulation OR de-population OR Demographic decline OR demographic anxiet* OR Fertility decline OR Second demographic transition OR Birth rate decline OR Declining birth rate OR Fertility decrease		AND	Subject headings: contraception, OR family planning OR abortion OR pregnancy termination OR pre-pregancy care OR Pre-conception OR assisted reproduction OR infertility therapy OR IVF OR reproductive health	
SEARCH 2	contracept* OR “family planning” OR abortion OR “pregnancy termination” OR “termination of pregnancy” OR “prepregan*” OR “pre-pregnan*” OR “preconcept*” OR “pre-concept*” OR “assisted reproduction” OR “infertility treatment” OR IVF OR “in vitro fertili?ation” OR “in vitro fertili?ation”	Adj5	Bans OR banned OR banning OR closing OR closure OR restrict* OR cut* OR defund* OR shutdown* OR reduction OR disrupt* OR downsiz* OR coercion	AND	pronatalis* OR pro-natalis* OR fertility incentive* OR population polic* OR low fertility OR declining fertility OR anti-abortion policies OR Population decline OR demographic crisis OR Sub-replacement fertility OR Below replacement fertility OR Shrinking population OR Ag?ing population OR population ag?ing OR depopulation OR de-population OR Demographic decline OR demographic anxiety* OR Fertility decline OR Second demographic transition OR Birth rate decline OR Declining birth rate OR Fertility decrease OR Post-transition*

Additionally, we will search for grey literature through Overton and Policy Commons databases. Also, we will review the reviews undertaken over the last 20 years to synthesise the data on what policies are to influence TFRs.

### Inclusion and exclusion criteria

The review will follow a structured approach to optimise search criteria and ensure consistent application of core concepts to guide the search. The Population/Participants, Concept and Context framework will be used to guide the review.^18^ Below is a summary of the inclusion and exclusion criteria in table 2.

**Table 2:** Inclusion and exclusion criteria

Domain	Inclusion	Exclusion
Population	Sexual and reproductive health (SRH) service users	Not SRHR service users
Concept	Pronatalist policies	Not pronatalist policies
Context	One of the tracers of SRH services: abortion, contraception, preconception care and fertility care.	Not in one of the tracers of SRHR services
Study design	All study designs	Case reports, expert opinions, editorials, literature reviews and retracted studies.
Time frame	Studies published between 1 January 1990 and 31 August 2025. This time frame ensures the search will capture the up-to-date studies.	Studies whose primary data are dated before 1990.
Language	Studies published in English	Studies not published in English

### Operational definitions

#### Sexual and reproductive healthcare services

This includes the following services (1) comprehensive sexuality education (in and out of school); (2) counselling and services for a range of modern contraceptives, with a defined minimum number of type of methods; (3) antenatal, childbirth and postnatal care, including emergency obstetric and newborn care; (4) safe abortion services and treatment of the complications of unsafe abortion; (5) prevention and treatment of HIV infection and other sexually transmitted infections; (6) prevention of, detection of, immediate services for and referrals for cases of sexual and gender-based violence; (7) prevention, detection and management of reproductive cancers, especially cervical cancer; (8) information, counselling and services for subfertility and infertility and (9) information, counselling and services for sexual health and well-being.

#### Pronatal policy

A government strategy designed to encourage a higher birth rate and population growth.

### Screening procedure

Two reviewers will independently screen titles/abstracts and full texts using pre-defined eligibility criteria above. Discrepancies will be resolved by consensus, and the reason for exclusion documented. If necessary, a third reviewer will be consulted to reach a consensus and agree on eligible studies for inclusion in the final evaluation. PRISMA flow diagram^17^ will be used to report and document the study selection outcomes.

### Data charting

A standardised electronic data extraction form will be developed. One reviewer will extract relevant information independently from identified studies using the predefined data extraction form. Data items will include basic study information (author, year of publication, study aim, study objective, study design and sample size, country/city), population type, disciplinary area, theoretical constructs used, area of SRH services, SRH measurements used, type of policy, rationale of policy, policy mechanisms, level of policy implementation, reported outcomes and rights implications (eight rights principles) and impact on gender and family norms. A second reviewer will validate 20% of the data extraction. In the event of disagreement, two reviewers will compare and discuss with each other; if necessary, a third reviewer will be consulted to reach a consensus.

### Quality assessment

Given the diversity of sources and study designs to be used, we will not conduct a critical appraisal of individual sources of evidence. Quality assessment is not mandatory for scoping reviews because the goal is to map the breadth of a topic, not to synthesise evidence for clinical practice or policy.^19^

### Data analysis and synthesis

Tables will be used to provide a summary of the data extracted from the evaluated studies. Findings from the studies will be synthesised by comparing the interventions across studies. It is expected that the review will be primarily descriptive, given the diversity of the policies. Categorisation schemes will be developed based on the data synthesis to categorise the policies, their dimensions and impacts on SRHR services. Using the data extracted, identified policies will be mapped and categorised to obtain their features of interest. The analysis will examine demographic characteristics of the population, such as regions, target age groups and gender, to explore potential discrepancies. Consistent with scoping review methodology, the extracted data will be synthesised using a descriptive and thematic approach. We will first present the results in table form, mapping the characteristics of included studies (eg, year, country, study design, population), as well as policies and impacts. This will allow for the identification of patterns, trends and gaps in the literature.

A narrative synthesis will accompany the table to highlight the breadth of the evidence, key concepts and areas of convergence and divergence across studies. Where appropriate, we will categorise findings according to thematic domains relevant to the research questions (eg, policy types, contextual factors). No critical appraisal or meta-analysis will be conducted, as the objective of this review is to provide an overview of the available evidence rather than to assess study quality or generate pooled effect estimates.

### Expert consultation

A draft of the review report will be developed and shared with an expert panel for comments and feedback to validate findings and assess implications. The expert panel will comprise a wide range of stakeholders, including researchers, practitioners, policymakers and community representatives.

## Ethics and dissemination

This study does not involve human participants. Contact details for ethics are available online (*ercsec@who.int).*

### Patient and public involvement

It was not appropriate or possible to involve patients or the public in the design, conduct, reporting or dissemination plans of our research.

### Discussion and potential implications

This scoping review aims to identify the extent and nature of the evidence on whether pronatal policies affect selected tracer SRHR services and to provide a snapshot of the evidence on the relationship between pronatalist policies and the four selected tracer sexual and reproductive health services. Key trends will be identified and empirical examples provided. These findings will be useful to assist all stakeholders to understand the potential impacts of proposed pronatal policies and to mitigate potential negative impacts.

## Supplementary material

10.1136/bmjopen-2025-115081 online supplemental file 1

## Data Availability

No data are available. All data relevant to the study are included in the article or uploaded as supplementary information.
