# Supplementary material for: Effects of pronatal policies on sexual and reproductive health services: a protocol for a scoping review
Source: BMJ Open. 2026 Apr 15;16(4):e115081. doi: 10.1136/bmjopen-2025-115081 (PMC13084901; doi:10.1136/bmjopen-2025-115081)
Supplement: Supplementary file 2 [file bmjopen-2025-115081.reviewer_comments.pdf]

PEER REVIEW HISTORY

BMJ Open publishes all reviews undertaken for accepted manuscripts. Reviewers are asked to complete a checklist review form and are provided with free text boxes to elaborate on their assessment. These free text comments are reproduced below.

ARTICLE DETAILS

Title (Provisional)

The effects of pronatal policies on sexual and reproductive health services: A protocol for a scoping review

Authors

Mburu, Gitau; Galati, Alanna; Chou , D; Ganatra, Bela; Kiarie, James; Boydell, Victoria

---

VERSION 1 - REVIEW

**Reviewer**	**1**
**Name**	**Kelly, Patricia J**
**Affiliation**	**Thomas Jefferson University**
**Date**	**24-Jan-2026**
**COI**	**None**

Excellent, timely topic with strong methods. Only one tiny comment--on page 7, line 23, please define/clarify "tracer" services.

VERSION 1 - AUTHOR RESPONSE

Response to reviews - bmjopen-2025-115081	
Excellent, timely topic with strong methods. Only one tiny comment--on page 7, line 23, please define/clarify "tracer" services.	Definition added and states: “tracer services, a subset of comprehensive SRHR services that can be used as proxy indicators to measure or assess the impact on overall SRH services.”
If you have selected ‘Yes’ above, please provide details of any competing interests.: Not applicable	Text changed as suggested.
